# Middle School Students' Experiences with Place-Based STEM Outreach

**DOI:** 10.1007/s11165-025-10245-1

**Published:** 2025-03-25

**Authors:** Tugba Boz, Nora Smith, Rebekah Hammack, Hilarie Davis, Jamie Cornish

**Affiliations:** 1 Department of Education, Health, and Behavior Studies, University of North Dakota, Grand Forks, ND USA; 2 Academic Technology and Outreach, Montana State University, Bozeman, MT USA; 3 Department of Curriculum & Instruction, Purdue University, West Lafayette, IN USA; 4 Technology for Learning Consortium, Stuart, FL USA

**Keywords:** Place-based education, STEM outreach, Health resources, Disease ecology

## Abstract

A five-day workshop, rooted in place-based approaches, was offered to 24 underserved/underrepresented middle school students. The workshop's activities centered around three key concepts of disease ecology: 1) the interactions between living things and their environment, 2) the impact of environmental interactions on health, and 3) the role of scientists and students in improving health. Throughout the week, students reflected on the disease ecology concepts in relation to their own lives and communities after each session. Daily reflections and student interviews were used to explore students' successes and challenges with the activities, as well as the relevance of place-based learning for their understanding of these three disease ecology concepts. The analysis revealed that the workshop was influential not just in teaching disease ecology to middle school students but also in fostering a deeper interest in science through hands-on learning and place-based activities. The connection to their places made the learning more relevant and interesting for students. The student reflections on each of the disease ecology questions showed that while students faced challenges in connecting disease ecology concepts to personal and community health practices, the overall trend indicated a positive trajectory in students' understanding and application of disease ecology topics. We conclude that the workshop demonstrated the effectiveness of combining place-based pedagogies in engaging underserved middle school students in disease ecology and its real-world implications.

## Background

Scholars have long established that informal and non-formal learning^1^ environments can yield positive impacts, such as increased student interest and confidence in STEM topics. Additionally, access to varied approaches to learning STEM increases chances that students will pursue a STEM career (Aikenhead, 2006; Roberts et al., 2018). Several scholars highlight the role of universities as essential providers of supplemental and non-formal learning opportunities for K-12 students (e.g., Bevan & Menezes, 2018; Hamilton, 2015). These opportunities allow students to engage with scientific communities and professionals and enhance their understanding of STEM fields. Research shows that non-formal STEM opportunities including university-based science outreach programs positively influence students’ understanding of the nature of science (Bell & Lederman, 2003). Furthermore, they increase awareness and enjoyment of science among students (Davies, 2008) and play a critical role in developing interest and influencing entrance into STEM majors (Wang, 2013). In this paper, we describe our STEM outreach workshop to engage middle school students with STEM activities, specifically focusing on disease ecology, in a week-long summer camp. In designing the activities, we adopted a broad definition of disease ecology. Disease ecology focuses on how species’ interactions and abiotic components of the environment affect patterns and processes of disease (Kilpatrick & Altizer, 2010). For this project, we define disease ecology in its broadest sense to look beyond the interactions between a single pathogen and host to explore all the factors responsible for infectious and environmental disease in human and animal populations. Our approach encompasses a multidimensional view of the social and ecological determinants of health. This comprehensive perspective integrates various aspects of disease ecology from disciplines such as medicine, microbiology, geography, nutrition, genetics, entomology, immunology, and epidemiology. In the design and implementation of the activities in the workshop, we also prioritized students' interests and cultural practices. Our approach emphasized place-based learning opportunities and cultural relevance, seamlessly integrating “STEM learning to learning within school, home and other settings” (Hamilton, 2015, p.1). As our workshop participants came from rural and Indigenous schools, we also prioritized Indigenous tribal histories and traditional ecological knowledge (Semken, 2005; Windchief, 2019) and emphasized the uniqueness of *rural places* (Aikenhead & Jegede, 1999) by encouraging participants to consider issues of health and well-being within their communities.

Furthermore, focusing on disease ecology in our activities, we aimed to bridge the gap in middle schoolers' understanding of related concepts and vocabulary, a gap that exists despite their firsthand experiences with health issues in their communities. This focus is underpinned by findings from Kafai et al. (2022), who conducted a scoping review on educational interventions for respiratory infectious diseases. Their review revealed a notable lack of such interventions, particularly for middle school students, with most existing studies aimed at high school students. By targeting this younger age group, our STEM outreach workshop addresses a critical gap to ensure that middle school students gain early insights into important health topics.

### Aim and Research Questions

In this paper, we aimed to explore how the particular workshop we designed and implemented contributed to students’ engagement with STEM, specifically with regard to science, and their increased understanding of disease ecology concepts and practices. We targeted the following research questions:

1. How do middle school students describe their engagement with place-based science lessons with a focus on disease ecology after the workshop?
2. How do place-based lessons enhance students' understanding of the interactions between living things, the environment, and health in the context of disease ecology over the course of the workshop?

### Theoretical Framework

Building on the rich tradition of *place-based* and *place-conscious* educational research, our workshop was inspired by Gruenewald's seminal exploration of the pedagogy of place in 2003, which served as a foundational pillar for our approach to connecting STEM learning with the immediate surroundings of students. Gruenewald (2003) argued that critical pedagogy and place should be converged into a critical pedagogy of place, which combines cultural decolonization from critical pedagogy and ecological *reinhabitation* from place-based education. Critical pedagogy, originally introduced by Paulo Freire (1996), argues that the educational system serves the needs of those in power and calls for the transformation of education to overcome the social structures that are oppressive to different groups of students. Place-based education is a pedagogical method that utilizes the local environment or community as the starting point for learning (Sobel, 2004). Through place-based learning students engage in hands-on experiential learning activities that connect them to their local environment. Gruenewald argued that combining aspects of critical pedagogy and place-based education can enhance education research by allowing for the connection of social and ecological factors. Gruenewald proposed (2003):

By promoting a pedagogy for student engagement in community life, place-based educators embrace aims beyond preparing students for market competition…like critical pedagogues, place-based educators advocate for a pedagogy that relates directly to student experience of the world, and that improves the quality of life for people and communities. (p.7)

In line with Gruenewald's vision, our approach to the design and implementation of the workshop emphasized the importance of cultural relevance and ecological sustainability. Culturally relevant teaching seeks to make learning experiences more efficient and relevant for traditionally marginalized students by connecting learning to students’ lived experiences and centering their local knowledge in classroom instruction (Gay, 2018). We encouraged students to consider how their learning could contribute to their well-being and the well-being of their community and the environment. By doing so, we aimed to move beyond traditional STEM education's focus on market competitiveness and towards a more holistic and inclusive approach that values the interconnectedness of people, place, and ecology. In addition to Gruenewald's insights, considering that our students largely come from rural and Indigenous schools, we also built our initiative on Avery's, 2013 insights into the crucial role of local knowledge in rural science education. Avery (2013) emphasized the importance of local knowledge in making STEM education relevant for students by connecting it to their local rural context, hence enhancing the learning outcomes of rural students in STEM subjects.

Research consistently demonstrates that place-based education programs significantly boost student motivation and engagement, particularly among those at risk of withdrawing from formal education (Avery, 2013; McInerney et al., 2011; Powers, 2004). Empirical evidence positions place-based science education as a highly effective method for engaging a diverse spectrum of learners in meaningful scientific inquiry and attracting underrepresented groups to science due to its relevance and inclusivity (Emekauwa, 2004). Additionally, beyond sustaining interest in science coursework, these programs enable students to preserve their connection to rural origins and communities (Bouchard & Wike, 2022) and foster a deep connection between hands-on, authentic experiences and the cultivation of a strong sense of community belonging, place, and active citizenship (Sobel, 2004).

Place-based education facilitates STEM learning and teaching with culturally relevant pedagogies, which recognize language, culture, and literacy as integral to Indigenous cultures and knowledge systems (Lee et al., 2015). Using place-based education as our framework, we highlighted the importance of *place* within Indigenous science education and Indigenous methodologies that emphasize *the centrality of place* in Indigenous knowledge systems (Bruchac, 2016; Peltier, 2021; Pineda et al., 2020; Smith, 2021; Windchief & San Pedro, 2019). To respect and integrate these Indigenous knowledge systems, it is important to create inclusive learning environments that honor students’ cultural backgrounds and identities in preparing them for active engagement in a global society.

## Description of the Workshop

### Context of the Workshop

In the U.S. state of Montana, the Office of Public Instruction—a government agency responsible for overseeing public education—reports that approximately 24,000 students in the 7th and 8th grades are currently enrolled in the state's public schools (Montana Office of Public Instruction, 2024a). Montana's approach to education is distinctive, as the state constitution mandates Indian Education for All, a living things policy that requires schools to integrate Native American—a term used to describe the Indigenous Peoples of the continental United States—history, culture, and perspectives into their curricula (Montana Office of Public Instruction, 2024b). With seven reservations and the headquarters of 12 federally recognized tribal nations—sovereign entities recognized by the U.S. government—Indigenous youth constitute about 11% of Montana’s school-age population. The Office of Public Instruction has acknowledged the urgent need to enhance students’ access to STEM (Science, Technology, Engineering, and Mathematics) experiences in Montana, as Montana ranks low in middle school science achievement and access. According to the 2022 State Report Card, only about 36% of students in the state are proficient in science at their grade level (Montana Office of Public Instruction, 2024c).

The workshop was conducted as part of a nationally funded project and took place during a one-week residential summer camp for underserved middle school youth in Montana in the United States. The camp was created to introduce youth to college and inspire them to consider science-related careers. Campers attended the same three workshops each day totaling 7.5 h of instruction time (1.5 h for five days). The camp sessions all covered science and technology related topics; however, our workshop was the only one that had sessions related to disease ecology.

### Content and Delivery of the Workshop

We designed the workshop to teach students about the factors responsible for infections and environmental disease in human and animal populations using place-based lessons. The three main concepts the workshop lessons focused on were 1. Living things interact with each other in the environment in good and bad ways. 2. How you interact with your environment can affect your health. 3. Scientists work to improve our health – and you can help too. The camp was residential and took place on the university campus. Undergraduate students from the university and instructors from a tribal college —a higher education institution primarily serving Native American communities— located in the region taught lessons to engage middle school youth in hands-on, place-based and culturally relevant activities. Instructors emphasized cultural relevance in a daily exercise asking youth to reflect upon how to connect their learning to their own health and their community. Students were given time to think about and write down how the daily lesson connected to their lived experience and local knowledge. Then, there was time every session to discuss their reflections. Each day of the workshop covered a specific topic (see Table 1) and the instructors taught the content to two groups of students, with each session lasting 90 min.

**Table 1:** Content of the workshop

Time	Topic	Learning Objectives
Day 1	Introduction to disease ecology and microbes	The student will identify that microbes are everywhere and can accurately differentiate between beneficial and harmful microbes
Day 2	Microbes and infections	The student will clearly distinguish between viruses and bacteria and explain how they can infect people and animals and can list illnesses and diseases affecting Montana's ecosystem and their transmission methods or implications
Day 3	Water and health – Exploring water systems in Montana and sources of pollution and collecting samples	The student will accurately determine pollutants in the state and explain the interaction between water particles and the environment
Day 4	Nutrition and Indigenous food sovereignty	The student will recognize the relationship between nutrition and the environment on health and explain the concept of Indigenous food sovereignty, using the example of Native food sovereignty in Montana and its historical significance
Day 5	Our home/community – Microplastics, water filtration, workshop summary	The student will evaluate growth in petri dishes and explain the presence and implications of microplastics in various environmental settings

On Day 1, students were introduced to the overall topic of disease ecology, with a focus on microbes. They examined prepared slides of plant and animal cells under microscopes and made hypotheses about which objects around them might be harboring the most microbes. Students prepared to test their hypotheses by swabbing samples from inside and outside the building onto agar plates, then setting them aside until Day 5 lesson. On Day 2, the topic was microbes and infections. Students identified microbes in Montana that are associated with diseases like Rocky Mountain Spotted Fever and Blue Tongue and microbes such as Coronavirus and E. Coli. Working in teams, they played a game using keys and locked boxes to discover the particular microbe that causes each disease, if the disease is contagious and how it spreads, and possible treatments for the disease. The students then presented their findings to the other groups. In a second activity, they reviewed case studies with photos, data, and a list of symptoms to identify locally found diseases (an example case study: “After hiking in the Bitterroot Valley, a 30-year-old male comes into the emergency room with a high fever and a spotted rash that started around his hands and feet. They think he may have been bitten by a tick"). On Day 3, the topic was water and health. Students collected samples from water sources on campus and learned about watersheds in Montana, waterborne microbes, sources of pollution, and pH and dissolved oxygen levels in water. Then they analyzed their own samples and hypothesized about the reasons for its condition. They also learned about the perspectives of Indigenous water advocates. On Day 4, the topic was nutrition and Native science. Students learned about how important food is to health through local Indigenous stories about food and health, studying nutritional labels (US and EU), and understanding taste through sampling different foods particular to Montana. Students explored food and culture in families and communities. As a way to bring all of the week’s topics together—microbes, infections, water, and food—on Day 5, the focus was on “our home/community.” Students analyzed their agar plates they swabbed on Day 1 and evaluated new water samples for microplastics and pollution. They learned about types and sources of microplastics, and how to identify and reduce them in the environment. Finally, they learned about local water pollutants and algal blooms and their impact on pH and dissolved oxygen.

## Methods

We used intrinsic case study research as our research design as the focus of the study was to understand the case of our disease ecology workshop in depth (Stake, 2023), and our study began “with the case already identified” (p. 127). By using case study research, we aimed to uncover the details of middle school students’ experiences with the summer camp workshop on disease ecology. Therefore, we utilized this research design for descriptive purposes without any intention to generalize the findings beyond this specific case examined (Stake, 2023; Yin, 2018). This approach allowed us to gain deep insights into the individual experiences and perceptions of the participants.

### Positionality Statement

As this study has a place-based and culturally relevant focus, we believe it is important for us to reveal our positionality in relation to the study (Holmes, 2020). Five authors drafted this paper. The first author self-identifies as Euroasian female. The rest of the authors self-identify as White American females. We all have lived or worked in rural places and have experience with STEM education at the middle school level. We have worked on educational-related research and evaluation projects and programs with Indigenous peoples and are committed to practicing cultural humility within learning contexts. We value local knowledge and recognize the importance of place in the construction of knowledge and meaning. Therefore, an understanding of place cannot be removed from our interpretation of the data. We acknowledge that our positionalities influenced the study to some extent and the way we conducted this study.

### Participants

24 students participated in the workshop at the summer camp, and we included all the participants in this study. The workshop had two sections, with 13 students in the first section and 11 students in the second section. The participants ranged from rising 6th through 9th graders (approximately 11 to 14 years old), with a balanced representation of both male and female students. Participants represented seven underserved communities from around Montana and consisted of 13 white participants, 11 Native American participants, and one Hispanic participant. All names and personal identifiers were replaced with pseudonyms to maintain confidentiality.

### Data Collection

We used two primary sources of data: student interviews and student-generated artifacts. Both data sources allowed us to carry out an in-depth investigation of our case of middle school students’ experiences with disease ecology (Stake, 2023). We conducted the interviews with all participants at the end of the workshop, and each interview was approximately 15–20 min in duration. We developed a semi-structured interview protocol to understand students' perspectives about and experiences with the workshop activities. Key questions included inquiries about their successes, challenges faced during the workshop activities, and the relevance of place-based specific information in the activities. Questions such as "What were you successful at in the activities?" and "Did you notice the activities had Montana information and examples? If yes, how and did that make you more interested in the activities?" were addressed during the interview.

We collected student artifacts in the form of written responses to three specific questions designed to inquire into their understanding of the activities on disease ecology. These questions were:

1. [Q1] In your own words, using examples from home and from camp, explain how living things interact with each other and the environment.
2. [Q2] In your own words, using examples from home and from camp, explain how all those interactions affect your health.
3. [Q3] In your own words, using examples from home and from camp, explain what you have learned about science and from your home community about staying healthy.

**Fig. 1:**
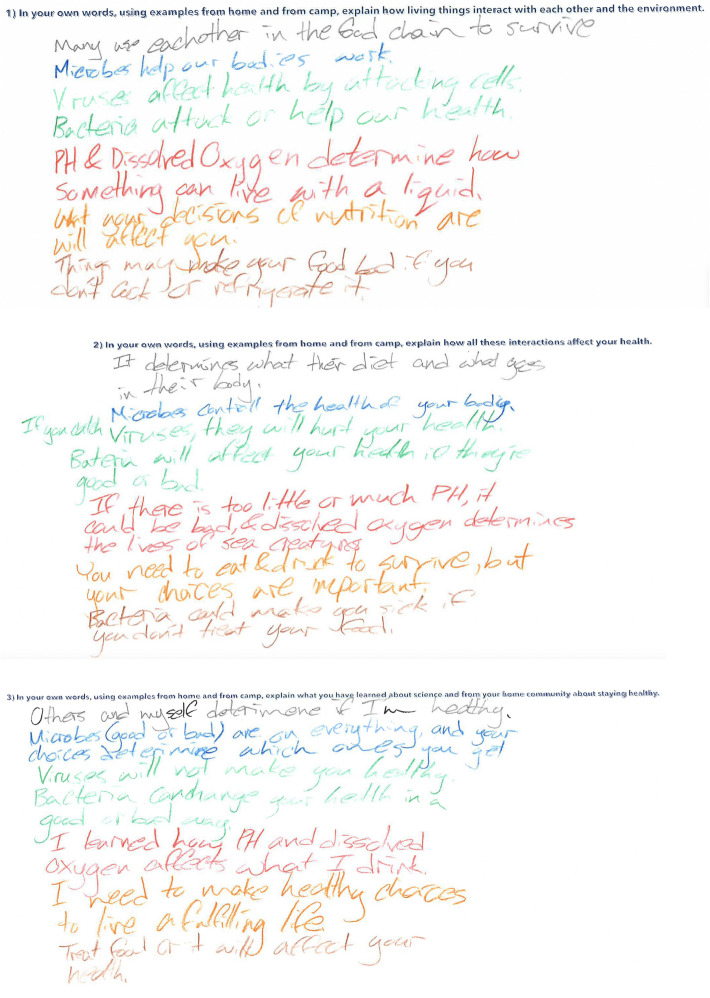
Example to student responses to three questions

Each question was printed at the top of an 11 in × 16 in piece of paper, and each student was given the papers for each question at the beginning of the sessions (as shown in Fig. 1). They were instructed to write their responses at the end of each day using a different colored pen to indicate the day of the response (blue for day 1, green for day 2, red for day 3, orange for day 4, brown for day 5). We provided guidance on the meaning of each question and encouraged students to reflect on what they learned in the workshop and how it applied to them.

### Data Analysis

We used a multi-method approach in analyzing student interviews and artifacts to ensure we fully understood the data. To analyze the interviews, we first obtained verbatim transcripts of the recordings. We used Atlas.ti (version 23.4), a qualitative data analysis software, to organize and manage the interview data. We conducted our analysis by iteratively coding and categorizing the interview data. For approximately three months, the first and second authors of this paper met weekly to collaborate on the analysis process. During these meetings, we initially conducted open coding to identify preliminary themes, patterns, and concepts emerging from the data (Saldaña, 2016). As the coding process progressed, we refined our categories and finalized our codebook (see Table 2) to establish the recurrent themes. Once the coding was complete, we engaged in a sense-making process of the entire coded interview data. When disagreements arose, we resolved them using various strategies. We discussed each disagreement in depth, presenting individual interpretations and specific examples from data, which led to a consensus on the coding decision that reflected a synthesis of our perspectives. When necessary, we refined our codebook, either by clarifying the descriptions or introducing new ones. This whole process helped us address sources of disagreement. Once we completed the process of coding, we grouped similar codes under analytical categories (Maxwell, 2013). For example, we identified codes such as "Fun and Hands-On," "Increase in Science Interest," "Comparing Camp to Regular Classroom," and "Teamwork." These codes, which reflected student engagement with the camp activities, were categorized under the "Engagement" category. For the complete list of codes and categories, please refer to Table 2 below.

**Table 2:** Codebook and categories

Categories	Codes
Engagement	Fun and Hands-On
	Increase in Science Interest
	Comparing camp to regular classroom
	Teamwork
Understanding	Increased understanding of disease ecology
	Different kinds of science and careers
	Feeling successful
	Connections to Montana
Feedback on Content	Water
	Lock Boxes
	Nutrition
	Bacteria/Viruses
	Challenges: Writing

In analyzing student responses to the given questions (see Fig. 1 above), we developed a rubric (see Table 3) based on the daily learning objectives set by instructors and researchers before the camp started. This rubric, as detailed in Table 3, was designed to evaluate the depth of student understanding as reflected in their responses and to enable valid judgment of student learning (Jonsson & Svingby, 2007). The criteria, corresponding to the learning objectives for each day, were used to assess the extent of evidence in student responses. We categorized the level of evidence into four levels: no evidence, minimal evidence, partial evidence, and full evidence. To illustrate how we used the rubric, consider Day 5: a score of 0 (no evidence) was assigned for responses that failed to evaluate growth in petri dishes or were unaware of microplastics. A score of 3 (full evidence) was given for responses that skillfully evaluated growth in petri dishes and recognized that microplastics are prevalent in the environment.

**Table 3:** Rubric

Days	No Evidence (score as 0)	Minimal evidence (score as 1)		
	Criteria	Criteria	Excerpt	Rationale
Day 1	Unable to identify microbes or understand their characteristics. OR incomplete/irrelevant/no response	Recognizes that microbes exist but lacks further details about them	“I could get sick, and possibly die.”	The student recognizes that microbes can cause illness, but does not provide further details about microbes in general
Day 2	Cannot differentiate between viruses and bacteria, or unaware of local illnesses and diseases in Montana. OR incomplete/irrelevant/no response	Has heard of viruses and bacteria but cannot elaborate on infection pathways or differences or has heard of some local illnesses or diseases but lacks detailed understanding	“Good bacteria interact with germs when you are sick.”	The student acknowledges bacteria, but the concept of "germs" is vague
Day 3	Lacks understanding of pollutants and their presence. OR incomplete/irrelevant/no response	Has heard of environmental pollutants but cannot elaborate on their nature or implications	“Living things, they'll share or use the same resource, that we use.”	The student shows general understanding of shared resources but is not directly addressing pollutants
Day 4	Unable to relate nutrition and environment to health. OR incomplete/irrelevant/no response	Has a basic idea about nutrition or the connection between nutrition and environment or has heard of Native food sovereignty in Montana	“Rainbow foods make us healthy.”	The student has a basic idea about diverse nutrition
Day 5	Cannot evaluate growth in petri dishes or unaware of microplastics. OR incomplete/irrelevant/no response	Has seen petri dish growth but is uncertain about why or how the growth happens or has heard of microplastics but lacks detailed knowledge	“Things may make your food bad if you don’t cook or refrigerate it.”	The student mentions petri dish growth/growth of microbes
Days	Partial evidence (score as 2)			
	Criteria	Excerpt	Rationale	
Day 1	Recognizes that microbes exist but has limited knowledge about their positive and negative roles	“Everything has microbes.”	The student recognizes that microbes exist everywhere but does not provide details about their roles, whether positive or negative	
Day 2	Recognizes basic differences between viruses and bacteria and their infection pathways but may have misconceptions about their infection pathways or knows a few local illnesses or diseases but lacks a comprehensive understanding	“If something bites another animal, it could affect its overall health by spreading whatever it had.”	The student recognizes transmission pathways but does not specify viruses or bacteria or any specific local disease	
Day 3	Recognizes that pollutants exist but may not fully grasp the interactions between water particles and the environment	“Fish rely on ppm in water.”	The student mentions ppm is related to water quality but lacks detailed understanding on how	
Day 4	Recognizes the impact of either nutrition or environment on health or has a vague understanding of Indigenous food sovereignty	“Plants can die if they don’t get what they need from the environment.”	The student emphasizes the environmental aspect but doesn't connect it directly to nutrition or Indigenous food sovereignty	
Day 5	Can observe growth in petri dishes, or has some understanding of microplastics (the effect of microplastics on the environment or health)	“Microbes can only work in certain temp.”	The student acknowledges how petri dishes work	
Days	Full evidence (score as 3)			
	Criteria	Excerpt	Rationale	
Day 1	Identifies that microbes are everywhere and can accurately differentiate between beneficial and harmful microbes	“Yellowstone Bluewater has bacteria that uses and it uses the sun for food.”	The student recognizes a specific type of beneficial microbe in Yellowstone (local ties) and its function. This shows a deeper understanding and differentiation	
Day 2	Clearly distinguishes between viruses and bacteria and explains how they can infect people and animals or can list illnesses and diseases affecting Montana's ecosystem and their transmission methods or implications	“For the rocky mountain fever, it can only spread it a tick BITES.”	The student identifies a specific local disease (Rocky Mountain spotted fever) and its transmission method	
Day 3	Can accurately determine pollutants in the environment and explain the interaction between water particles and the environment	“If the water has under 5 ppm of dissolve oxygen, then less animals can live in it.”	The student accurately connects dissolved oxygen levels to animal life and the quality of water	
Day 4	Recognizes the relationship between nutrition and the environment on health and explains the concept of Indigenous food sovereignty in Montana and its historical significance	“Hunting kills deers so that people can eat the dear so they get more nutrition.”	The student mentions a practice that can relate to Indigenous food sovereignty and connects hunting to nutrition	
Day 5	Can evaluate growth in petri dishes and explain that microplastics are everywhere in the environment	“Fish eat microplastics, then other organisms eat them, which then ends up on your plate.”	The student demonstrates an understanding of the food chain implications of microplastics	

We used the rubric for descriptive purposes only; in other words, by using the rubric for descriptive purposes, we were able to provide a clear and comprehensive overview without making inferential or causal assumptions. To ensure the accuracy and consistency of scoring, the first and second authors of the paper held several meetings to refine and discuss each score for each category in the rubric. During these meetings, any conflicts or discrepancies in scoring were addressed through thorough discussion. Any disagreements were resolved by re-examining the data and rubric criteria together until both authors reached a 100% agreement on each score (Stemler, 2004).

## Findings

### Students’ Descriptions of their Engagement with the Workshop

In describing their understanding of disease ecology and the activities/topics covered in the workshop, almost all students shared that they felt they learned new information during the camp, saying things like “I learned so much more than I thought I would” and “I learned a lot more about…” In addition to these more general statements, students also described some of what they learned in more detail, such as one participant who talked about water:

“So the water (activity) was pretty cool because I never knew so many things grew in water. And how much you actually need to filter it and make sure it’s good to drink. Yeah, otherwise it could make you really sick. Yeah, yeah…it was definitely different than what I’m used to. But pretty fun. Looking at how I’m, how much pH is in water and the dissolved oxygen, which I didn’t even know was in water.”

This statement shows that the student was thinking about the quality of water in a way that they had not previously. It also reflects that the student gained a deepened understanding of key water quality indicators.

In addition to learning new information related to disease ecology, the majority of the students spoke about the connections they saw between what they learned and protecting their own health and that of their families. This indicates not only an increase in awareness but also a potential shift towards more health-conscious behaviors. “So I learned a lot more about disease ecology…And I personally liked doing the activities about disease ecology because now I know how to prevent and help get rid of them if I do.” One student said, “I like learning about them. They’re fun to learn about and we can learn to stay safe from that, not to get sick,” and another described, “I learned a couple more diseases and how to stay safe from them. What to use if we get sick.” Others talked about how they could apply what they learned beyond just themselves. For example, one participant said they could “share [what I learned] with my family,” while another spoke about using the science learned to “help the environment around me.”

In reflecting on their engagement with the workshop, all students enthusiastically reported that the hands-on nature of the activities significantly enhanced their engagement and enjoyment. This enthusiasm is captured in one student's response during the interview. The student highlighted the distinctiveness of the interactive approach taken in the workshop compared to their usual classroom experiences:

“I liked what we did. It was pretty fun. I like that, I’m like, how much like how hands-on we were. Because like in, like in my other class…we don’t really do a whole lot, like activity wise. So I like this more hands-on and we got to do more.”

The workshop's hands-on activities sparking an increased interest in science was a common theme among multiple participants. For instance, one student articulated how the workshop transformed their apathy towards science into a genuine curiosity in topics like disease ecology, germs, and microplastics, which were covered during the workshop:

"I wasn’t like a really big fan of science and I wanted to like learn more about it. And [disease ecology workshop] definitely helped me like science better. Like, I’m more interested in the science which, like behind the like germs and microplastics and what they can do to you. Like before [disease ecology workshop] I wasn’t really like interested in that kind of stuff."

Another participant expressed how this workshop experience was different from their formal school experience that limited science to textbooks and worksheets:

"If it was like [on] a one to ten [scale], when I came here it was like a two. Now, I’m like at a six. Yeah…because it’s just really fun, just learning all these things…like science before (in school) because we just had to get to like a lot of papers. Yeah, this (disease ecology workshop) was hands-on."

Beyond the hands-on aspect, some students also valued the workshop's emphasis on local connections. Recognizing the relevance of what they were learning to their immediate surroundings made the educational experience more compelling. One student noted, "It’s just like where I live in, I think it’d be fun. And it’s always better to learn about where I live." This was echoed by another student who found that the local focus not only heightened their interest but also provided tangible examples they could observe in their environment, thereby enhancing the relevancy and impact of their learning:

"Yeah, [the local connection] increased my interest…because like, I mean, you can actually go and see those things and like, remember, oh, that’s bad for you, don’t do that. Or, like stuff like that. I mean, it’s definitely more interesting to me than like learning about other places in the world."

For this student, being able to “go and see” what they learned in action in their local environment made the learning more relevant and interesting.

Students also shared their perspectives on the activities, and student feedback on the activities varied, with different students preferring different activities. Most students spoke positively about all of the activities, frequently referencing the hands-on nature of activities as a positive quality. However, across all of the activities, there were positive comments and also negative comments for each activity. For example, when describing the water analysis activity, one participant stated, “I don’t like doing [water testing]” while other students said “it was an amazing class” and “[testing] the water was pretty cool.” This is not surprising as there are bound to be different interests across a group of diverse adolescents.

Some participants described writing as their least favorite portion of the workshop. Comments during the interviews such as “usually, I like more speaking, and like, I don’t really like just like writing stuff down,” and “We need less writing. Less writing, maybe more talking. Less individual, maybe like more conversation” were common. Additionally, camp instructors noticed that students struggled when they were asked to record answers to the big three questions. Each day, instructors served as scribes for students who asked for help with writing their ideas on paper. This practice showed the necessity of developing diverse methods, including digital tools, to empower students to express their understanding in multiple ways during the workshop.

Overall, the interviews show that the workshop was influential in not just teaching disease ecology to middle school students but also in fostering a deeper interest in science through hands-on learning and place-based culturally relevant activities.

### Students' Understanding of the Interactions Between Living Things, the Environment, and Health

We used student artifacts to explore students' understanding of (Q1) how living things interact with the environment, (Q2) how these interactions affect health, and (Q3) what they have learned about staying healthy. The results of the scoring show that on different days and with different activities, students’ understanding of disease ecology concepts varied.

**Fig. 2:**
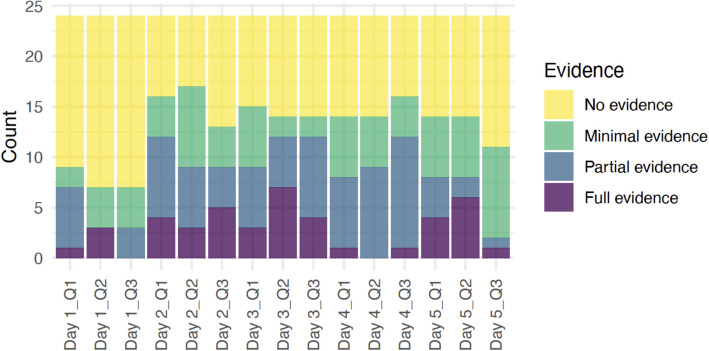
Stacked bar chart of evidence level counts for each day and question

On the first day, students were introduced to the basics of disease ecology, focusing on microbes. They examined prepared slides of plant and animal cells under microscopes and made hypotheses about which objects around them might harbor the most microbes. Day 1 aimed to teach students how living organisms interact with their environment on a microscopic level [Q1], identify potential hotspots for microbial life—how diseases can spread [Q2], and the importance of hygiene and sanitation [Q3]. As shown in Fig. 2, more than ten students displayed a lack of understanding across all three questions. Fifteen students demonstrated no evidence of understanding the interactions of living things with each other and the environment, and similarly, 17 students were unclear on how these interactions affect health and what they had learned about staying healthy. Only a few students showed full evidence of understanding, with one student for Question 1 [Q1], three students for Question 2 [Q2], and none for Question 3 [Q3].

On Day 2, the lesson shifted its focus to infections, a crucial component of disease ecology. Students identified microbes in Montana associated with diseases like Rocky Mountain Spotted Fever, Blue Tongue, Coronavirus, and E. Coli. Day 2 aimed to teach students the specific relationships between pathogens and their hosts [Q1], identify the specific diseases that affect people’s health in Montana [Q2], and recognize the necessity of local disease awareness for public health [Q3]. This day was more interactive and included many hands-on activities. On Day 2, four students demonstrated full evidence of understanding, and eight showed partial evidence. We found these numbers promising and can claim that the interactive activities of Day 2 might have helped students better grasp the complex relationships between pathogens and their hosts. In response to Question 2 on Day 2, three students showed full evidence. Six students showed partial evidence, and five students showed full evidence. For Question 3 on Day 2, we observed a similar pattern with Day 1. Compared to Question 1 and 2, there were less students showing full and partial understanding of staying healthy in the presence of microbes and infections.

On Day 3, students explored sources of water pollution and collected water samples on campus. Day 3 aimed to teach students the relationships between water sources and the organisms living in them, and the sources of water pollution [Q1], how water quality is important for health and the impact of water pollution on the environment and health [Q2], and preventive measures and solutions to water pollution, including Indigenous perspectives [Q3]. For Question 1 on Day 3, nine students showed no evidence of comprehension; six demonstrated minimal evidence; six displayed partial evidence; and three exhibited full evidence. This distribution suggests that a significant number of students still struggled with the concept. In response to Question 2 on Day 3, the results were slightly more skewed towards higher levels of understanding, with seven students showing full evidence of comprehension. Question 3 on Day 3 showed a similar trend to Question 1, with a moderate distribution of responses. These results on Day 3 suggest that the hands-on activity of collecting water samples might have made the implications of water pollution tangible and understandable for the students, and they were beginning to understand the causes and effects of water pollution. We also observe in the results that applying this knowledge to propose solutions or preventive health measures was still challenging for some.

Day 4 introduced nutrition and Native/Indigenous science. Day 4 aimed to teach students the relationship between nutrition and health from a Native/Indigenous science perspective [Q1], the impact of traditional dietary practices on health [Q2], and the application of Native/Indigenous science in modern health practices [Q3]. For Question 1 on Day 4, ten students demonstrated no evidence of understanding; six showed minimal evidence; seven displayed partial evidence; and only one exhibited full evidence. In response to Question 2 on Day 4, the results were somewhat mixed. Ten students showed no evidence of comprehension; five demonstrated minimal evidence; nine displayed partial evidence; and none exhibited full evidence. Question 3 on Day 4 showed eight students demonstrating no evidence of understanding, four showing minimal evidence, eleven displaying partial evidence, and only one exhibiting full evidence. The results for Day 4 showed that the majority of students engaged with the cultural perspectives in nutrition and health at a deeper level to some extent, and some of them found it somewhat difficult to connect traditional dietary practices with health outcomes.

On Day 5, students were introduced to microplastics and water filtration and included a summary of the entire week. Day 5 aimed to teach students the issue of microplastics in the environment [Q1], water filtration techniques and their effectiveness in removing contaminants like microplastics [Q2], and effects of microplastics and all the topics covered in the week on community health [Q3]. For Question 1 on Day 5, ten students showed no evidence of understanding; six demonstrated minimal evidence; four displayed partial evidence; and four exhibited full evidence. In response to Question 2 on Day 5, ten students showed no evidence; six demonstrated minimal evidence; two displayed partial evidence; and six exhibited full evidence. This indicates that the hands-on activity of exploring water filtration methods might have helped students better understand the practical aspects of addressing water pollution. For question 3, we saw thirteen students demonstrating no evidence of understanding, nine showing minimal evidence, one displaying partial evidence, and one exhibiting full evidence. This suggests that synthesizing the week's learning into a coherent understanding was challenging for many students, although a few were able to make meaningful connections.

Considering all the responses to each question, students continued to show varying levels of comprehension across different disease ecology concepts over the week.

In particular, students' responses to Question 3 throughout the week suggest that connecting disease ecology to personal and community health practices was challenging for students. The interactive and hands-on activities, especially on Days 2 and 5, seem to have played a significant role in enhancing students' comprehension of the complex relationships between microbes, the environment, and health. The integration of cultural perspectives on Day 4 also contributed to a deeper engagement with the material, although it remained a challenging concept for some students. In conclusion, the week-long workshop on disease ecology and microbes demonstrated the importance of interactive and culturally relevant teaching approaches in facilitating student learning. While challenges remain in fully grasping the interconnectedness of disease ecology concepts and the connection to health practices, the overall trend indicates a positive trajectory in students' understanding and application of these important topics.

## Discussion and Implications

The findings from the disease ecology workshop for middle school students highlight several key points regarding student engagement and learning. Our approach, inspired by Gruenewald's (2003) critical pedagogy of place and Avery’s local rural knowledge (2013), emphasized local connections and cultural relevance. By situating disease ecology within the context of students’ environment and community, we aimed to foster a sense of connectedness and relevance for students’ learning of disease ecology (Sobel, 2004). Our findings show that this approach increased students' interest in science and enabled them to see the practical implications of disease ecology in our week-long workshop. Moreover, students expressed a greater appreciation for the science that directly affects their communities. This finding also reinforces studies in the literature suggesting that STEM outreach programs can help increase students’ interest in STEM (e.g., Gossen et al., 2021) and provide them with an open space to make connections with real-world science in their immediate environment.

Our findings show that presenting place-based activities in a hands-on and interactive manner was meaningful in engaging students and fostering a deeper understanding of disease ecology concepts. In particular, student interviews suggest that students appreciated the interactive nature of the activities in the workshop that facilitated their learning compared to their formal school experience and science lessons that heavily rely on textbooks and worksheets. This preference aligns with the findings in the literature (e.g., Baran et al., 2016).

However, our findings also revealed that students had challenges in attaining certain objectives of the workshop. In particular, students faced difficulties in making inferences about health from the disease ecology concepts. This indicates a pressing need for more focused and explicit instruction in making connections between disease ecology concepts and health. More broadly, it highlights the importance of integrating scientific concepts with real-world contexts to enhance student comprehension and application skills.

Furthermore, many students expressed a preference for more conversational and interactive learning experience over written activities in the workshop. Such a finding is also expected considering that the majority of students in the workshop were from Indigenous tribes where oral tradition and storytelling play a significant role in knowledge transmission and learning (Smith, 2021). In these cultures, learning is often communal and interactive, and there is a strong emphasis on listening and speaking. Students’ preference for conversational and interactive learning experience observed in the workshop reflects this cultural background (Lee et al., 2015). In addition, the preference for more discussion and verbal engagement in the camp workshop might indicate that students in non-formal learning environments may have different expectations for engagement compared to traditional classroom settings. Research also suggests that these environments often provide a more relaxed and flexible setting, which can encourage students to express themselves and explore STEM in depth without the constraints of formal education (e.g., Bell et al., 2009; Kitchen et al., 2018).

In conclusion, by connecting STEM learning to students' local environments and cultural backgrounds, we created engaging and meaningful learning experience for our participant students. This approach contributes to the broader goal of making STEM education more accessible and relevant to diverse student populations. We also recognize that, in response to our findings, we can propose several recommendations for educators and program developers aiming to implement similar programs. It is essential to develop targeted lessons that explicitly bridge disease ecology concepts with local health implications. Examples from community health issues can be provided in introducing disease ecology concepts. Furthermore, given the strong oral tradition in Indigenous cultures, integrating local stories related to disease and health that resonate with students' cultural practices can be particularly effective. Reflecting on the preference for conversational and interactive learning over written activities in outreach programs, future programs should emphasize more collaborative group work and numerous interactive learning opportunities.

### Limitations

The analysis of student artifacts and interviews revealed some discrepancies. Student interviews showed more positive outcomes, and we had difficulties obtaining in-depth written responses or artifacts from students. In addition to students' expectations of less writing and more interaction and verbal engagement in informal learning environments, this discrepancy also highlights the importance of incorporating diverse data collection methods when researching non-formal STEM education programs (Hammack et al., 2023). Including data collection methods that focus on audio and video data might better fit the research conducted in these environments to gain a more comprehensive understanding of student learning and engagement. Therefore, the types of data used in this study can be considered a major limitation. Another limitation is the approach we took in analyzing student artifacts. In future studies, student responses to our three questions could be analyzed through coding and categorizing, in addition to using scoring rubrics.

## Data Availability

Data is not available due to the nature of the research.

## Footnotes

1 Informal learning is typically defined as learning through experience and without predetermined objectives or structured formats. Non-formal learning is characterized as organized learning outside the formal educational system, yet it possesses clear objectives (Werquin, 2010).
